# Effects of Thyroid Hormones on Lipid Metabolism Pathologies in Non-Alcoholic Fatty Liver Disease

**DOI:** 10.3390/biomedicines10061232

**Published:** 2022-05-25

**Authors:** Chia-Jung Liao, Po-Shuan Huang, Hui-Tzu Chien, Tzu-Kang Lin, Chau-Ting Yeh, Kwang-Huei Lin

**Affiliations:** 1 Graduate Institute of Biomedical Sciences, College of Medicine, Chang Gung University, Taoyuan 333, Taiwan; l329735@ms49.hinet.net (C.-J.L.); leo_6813@msn.com (P.-S.H.); 2 Department of Nutrition and Health Sciences, Chang Gung University of Science and Technology, Taoyuan 333, Taiwan; htchien@mail.cgust.edu.tw; 3 Research Center for Chinese Herbal Medicine, College of Human Ecology, Chang Gung University of Science and Technology, Taoyuan 333, Taiwan; 4 Neurosurgery, Fu Jen Catholic University Hospital School of Medicine, Fu Jen Catholic University, New Taipei City 242, Taiwan; tklin100@cgmh.org.tw; 5 Liver Research Center, Chang Gung Memorial Hospital, Linkou, Taoyuan 333, Taiwan; chauting@cgmh.org.tw; 6 Department of Biochemistry, Chang Gung University, 259 Wen-Hwa 1 Road, Taoyuan 333, Taiwan

**Keywords:** thyroid hormone, lipid metabolism, non-alcoholic fatty liver disease (NAFLD)

## Abstract

The typical modern lifestyle contributes to the development of many metabolic-related disorders, as exemplified by metabolic syndrome. How to prevent, resolve, or avoid subsequent deterioration of metabolic disturbances and the development of more serious diseases has become an important and much-discussed health issue. Thus, the question of the physiological and pathological roles of thyroid hormones (THs) in metabolism has never gone out of fashion. Although THs influence almost all organs, the liver is one of the most important targets as well as the hub of metabolic homeostasis. When this homeostasis is out of balance, diseases may result. In the current review, we summarize the common features and actions of THs, first focusing on their effects on lipid metabolism in the liver. In the second half of the review, we turn to a consideration of non-alcoholic fatty liver disease (NAFLD), a disease characterized by excessive accumulation of fat in the liver that is independent of heavy alcohol consumption. NAFLD is a growing health problem that currently affects ~25% of the world’s population. Unfortunately, there are currently no approved therapies specific for NAFLD, which, if left uncontrolled, may progress to more serious diseases, such as cirrhosis or liver cancer. This absence of effective treatment can also result in the development of non-alcoholic steatohepatitis (NASH), an aggressive form of NAFLD that is the leading cause of liver transplantation in the United States. Because THs play a clear role in hepatic fat metabolism, their potential application in the prevention and treatment of NAFLD has attracted considerable research attention. Studies that have investigated the use of TH-related compounds in the management of NAFLD are also summarized in the latter part of this review. An important take-home point of this review is that a comprehensive understanding of the physiological and pathological roles of THs in liver fat metabolism is possible, despite the complexities of this regulatory axis—an understanding that has clinical value for the specific management of NAFLD.

## 1. Thyroid Hormones and Action

### 1.1. Thyroid Hormones

Thyroid hormones (THs) are amino acid-derived hormones formed by the addition of iodine to thyroglobulin, the tyrosine component of a glycoprotein, that influence the growth, development, and metabolism of vertebrates. The production of THs is regulated by the hypothalamic–pituitary–thyroid (HPT) axis [1]. Specifically, thyroid-releasing hormone (TRH), released by the hypothalamus, stimulates the anterior pituitary gland to release thyroid-stimulating hormone (TSH), which, in turn, stimulates the thyroid to synthesize and release THs. A negative feedback loop is present in the HPT axis such that THs act on the pituitary and hypothalamus to inhibit TSH production. There are two main forms of THs secreted by the thyroid gland: T4 (3,5,3′,5′-tetraiodothyroxine), or thyroxine, the major product and precursor form of THs, and 3,5,3′-triiodothyronine (T3). T3, most of which can be generated in peripheral tissues by deiodination of T4 by iodothyronine deiodinases (DIO), is considered the potent active form. There are three main types of DIO (DIO1-3) in humans: DIO1 and DIO2 are responsible for the activating conversion of T4 to T3, whereas DIO3 converts T4 and T3 to the inactive metabolites reverse T3 (rT3) and rT2, respectively [2]. Therefore, modulation of the activity of DIOs and tissue-specific expression of DIOs can both positively and negatively regulate the functions of THs.

### 1.2. TH Actions

Canonically, THs act as ligands for TH receptor α (TRα) and TRβ encoded by *THRA* and *THRB* genes, respectively. TRs belong to the nuclear receptor superfamily, whose members can either induce or suppress gene expression by forming heterodimers with another nuclear receptor, retinoid X receptor (RXR), and binding to the TR-response element (TRE) on the promoter of target genes. Besides, TRs bind to promoters of target genes as monomer or homodimer, however, the binding is not as stable as the heterodimers of TR and RXR [3,4]. Notably, TRs regulate downstream target both in the presence or absence of THs. In the absence of THs, nuclear receptor corepressor 1 (NCoR1) and NCoR2 (also known as silencing mediator of retinoid and thyroid hormone receptors [SMRT]) serve as corepressors which are recruited to TR-RXR heterodimers bound to the TRE to prevent gene transcription. Further, in the presence of THs leads to conformational change in TRs and results in releasing of corepressors and recruiting of coactivators, such as SRC (steroid receptor coactivator) and CREB-binding protein/p300, and the target genes are turned on. T3 is the main active ligand for TRs because of the affinity of T3 is about 10-fold higher than that of T4. Binding of THs to TRs to transcriptionally regulate gene expression is considered the canonical pathway of TH action. However, some effects stimulated by THs occur within minutes, indicating that THs can act without directly binding the regulatory domain of target promoters, that is, though noncanonical pathways [5]. Flamant et al. precisely defined four types of TH action [6]. The canonical pathway is defined as type 1 action. Type 2 action refers to indirect binding of TH-TRs to DNA via other DNA-binding proteins, such as activating protein-1 [7], cyclic AMP response element-binding protein [8], and nuclear factor-kapa B [9], to interfere with the activity of THs in regulating the expression of genes. The third type of TH action corresponds to regulation of kinase pathways by TH-TRs through phosphorylation, rather than direct or indirect regulation of transcription. For example, phosphatidylinositol 3-kinase (PI3K) and mitogen-activated protein kinase (MAPK) pathways have been demonstrated to be phosphorylated and activated by TH-TRs [10,11]. Finally, type 4 action describes the signaling of THs through TR-independent pathways. Integrin αVβ3 has been identified as one such TH-responsive receptor [12,13]. For more details on TH and TR molecular mechanisms, refer to our previous publication [14].

## 2. TH and Lipid Metabolism in the Liver

### 2.1. Lipid Metabolism in the Liver

**Figure 1:**
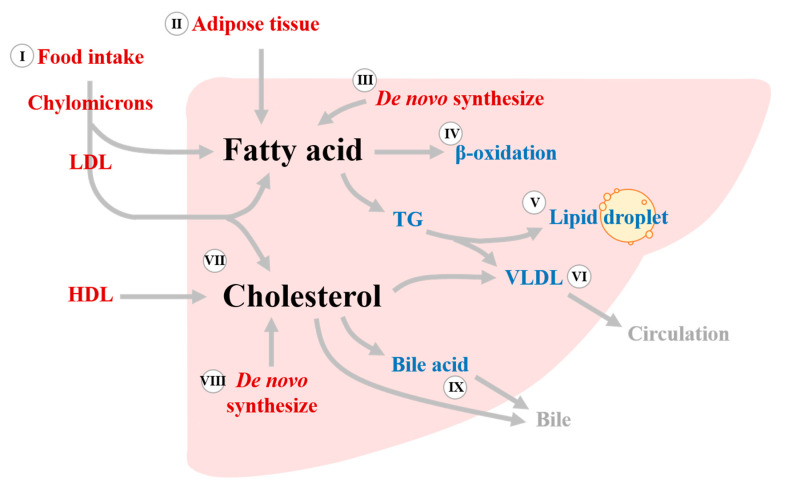
Lipid metabolism in the liver. The liver is the main site of fatty acid and cholesterol metabolism. Fatty acids come from three main sources: food intake (I), adipose tissue lipolysis (II), and de novo lipogenesis (III). Fatty acids are then used for the production of energy via β-oxidation (IV), synthesis of TGs and storage in lipid droplets (V), or packing into VLDLs and secretion into the circulation (VI). Hepatic cholesterol comes from circulating lipoproteins (LDL and HDL, VII) or de novo synthesis in the liver (VIII). Cholesterol is subsequently used as a component of the plasma membrane of hepatocytes, packaged into VLDLs and released as a cholesterol source for extrahepatic organs (VI), or directly released or converted to bile acid and released into the bile duct (IX). TG, triglyceride; VLDL, very low-density lipoprotein; LDL, low-density lipoprotein; and HDL, high-density lipoprotein.

The liver plays crucial roles in lipid metabolism, including acquisition of lipid, lipid storage, and lipid consumption. Lipids metabolized in the liver can be roughly divided into two types: fatty acid and cholesterol. There are two main sources of fatty acid that are sent to the liver for metabolism. The first is chylomicrons, which are derived from the intestine following digestion and absorption of lipid from food intake. The second is fatty acids released from adipose tissue by lipolysis. In addition to the uptake of fatty acids from extrahepatic sources, de novo lipogenesis (DNL) also occurs in the liver when excess glucose accumulates. After uptake or synthesis by the liver, fatty acids can be used for energy production via β-oxidation in mitochondria or peroxisomes, triglyceride (TG) synthesis and storage in lipid droplets, or TG secretion into the circulation in the form of very low-density lipoproteins (VLDL) as source of cholesterol for peripheral tissues. Cholesterol is another type of lipid metabolized mainly in the liver. Approximately 30% of cholesterol comes from food intake, whereas 70% comes from de novo synthesis, mainly in the liver. The fates of cholesterol in the liver include conversion to bile acid, packing in lipoproteins and secretion into the bloodstream, and use as a plasma membrane component. Hepatic lipid metabolism is summarized schematically in Figure 1.

### 2.2. THs and Lipid Metabolism in the Liver

The liver is one of the major target organs of THs, reflecting the high expression of TRs, especially TRβ, in this organ. Reports of TH effects on lipid metabolism have been documented as early as the end of the 19th and early 20th centuries [15]. Initially, it was noted that reversible hyperlipemia, with elevated levels of plasma total cholesterol (TC) and low-density lipoprotein cholesterol (LDL), were frequently observed in patients with hypothyroidism [16,17]. It was subsequently found that blood lipids were regulated by THs in patients with hypothyroidism who underwent thyroid replacement therapy [18,19,20], and shown that treatment with THs significantly reduce plasma cholesterol and lipoproteins. Furthermore, THs action through TRβ to regulate hepatic lipid metabolism was demonstrated in mice carrying a dominant-negative mutation in Thrb, the Thrb^PV/PV^ mice [21]. Decreased fatty acid β-oxidation and activated peroxisome proliferator-activated receptor-γ (PPAR-γ) due to dominant-negative effects of Thrb^PV/PV^ on TRβ contribute to hepatic lipid accumulation and steatosis. Collectively, these studies have established the critical roles of THs in lipid metabolism. The mechanisms by which THs affect lipid metabolism processes in the liver are summarized and described below.

#### 2.2.1. Regulation of Fatty Acid Uptake by THs

In general, the liver takes up about 25% of total free fatty acids from the blood flowing through it [22], a process mediated and regulated by translocases and transporters on the membrane of hepatocytes. Adipose differentiation-related protein (ADRP, also known as adipophilin, perilipin-2), fatty acid transporter proteins (FATPs), fatty acid translocase (FAT, also known as CD36), and fatty acid-binding proteins (FABPs) are the transporters and translocases responsible for fatty acid uptake by the liver [23]. A study in hypothyroid rats showed that fatty acid uptake in the liver is decreased relative to that in euthyroid rats [24]. Decreased hepatic FABPs and FAT expression have been demonstrated in rats with postnatal hypothyroidism [25]. In addition, another study in rats demonstrated that T3 inhibits fatty acid transport and utilization by the liver by decreasing the expression of liver FABP [26]. Collectively, these studies indicate that THs can regulate lipid metabolism by affecting fatty acid uptake through regulation of the expression of transporters or translocases. In addition to regulation of liver FABP by THs, it has been demonstrated that adipocyte FABP increases thermogenesis in brown adipose tissues by promoting the conversion of inactive T4 to active T3 [27]. It would be interesting to determine whether a positive feedback mechanism of FABP on THs operates in the liver.

#### 2.2.2. De Novel Lipogenesis (DNL)

In addition to promoting uptake of exogenous fatty acid, excess glucose can stimulate DNL in the liver. Glucose entering the glycolytic pathway is converted to pyruvate, which is then transported to mitochondria through the mitochondria pyruvate carrier (MPC). After the action of dehydrogenase and subsequent condensation with oxaloacetate (OAA), pyruvate forms citrate, which can enter the tricarboxylic (TCA) cycle to produce energy. Alternatively, if the cell has excess energy, citrate can be sent from mitochondria via the citrate carrier (CiC) to the cytoplasm, where it is reconverted to OAA and acetyl-CoA by ATP citrate lyase (ACLY). Acetyl-CoA, a substrate for DNL, is then catalyzed in turn by two critical enzymes, acetyl-CoA carboxylase (ACC) and fatty acid synthase (FAS). Many of the above-mentioned key factors that participate in DNL have been shown to be regulated by THs. Decreased expression of MPC and CiC is observed in hypothyroid rats, an effect opposite that observed in rats with hyperthyroidism [28,29,30,31]. ACYL expression is decreased in cultured primary hepatocytes isolated from thyroidectomized rats and is restored by treating cells with T3 [32]. In rats with propylthiouracil-induced hypothyroidism, ACYL is significant decreased relative to that in euthyroid rats [33]. ACYL is transactivated by sterol regulatory element-binding protein-1 (SREBP-1) [34], a member of the SREBP family of important lipogenic transcription factors. Although SREBP-1 is a bone fide TH-regulated gene [35,36], some contradictory results have been reported. For example, in one study using a mouse model, the authors demonstrated that T3 acts through a negative TRE on the promoter to directly inhibit SREBP-1 expression [35], whereas another study in human HepG2 cells reported that T3 activates SREBP-1 via non-genomic actions [36]. In addition, T3 induces SREBP-1 expression and also increases the activated form of SREBP-1 in chick embryo hepatocytes; this increased SREBP-1 acts in conjunction with TR to regulate ACC transcription [37]. FAS, another critical lipogenic enzyme, shares the same SREBP-1- and TH-independent or coordinated regulatory mechanisms as ACC [33,38,39].

#### 2.2.3. β-Oxidation

β-oxidation is the process that take place in mitochondria by which fatty acids are broken down to produce energy. In the liver, the release of fatty acids from TGs is mainly executed by hepatic lipase (HL) and adipose triglyceride lipase (ATGL). Levothyroxine, a TH replacement, significantly induces HL in individuals with subclinical hypothyroidism [40]. T3 induces recruitment of ATGL to lipid droplet (LD) surfaces to promote the release of lipids from LDs and entry into the β-oxidation pathway [41]. Transporting of fatty acids, especially long-chain fatty acids, from the cytosol to mitochondria is mediated by carnitine palmitoyl acyl-CoA transferase 1 (CPT1), the rate-limiting enzyme in β-oxidation. CPT1 converts fatty acyl-CoA to acylcarnitine, which can be transported across the inner mitochondria membrane via carnitine translocase (CAT). Once in mitochondria, acylcarnitine can be converted back to fatty acyl-CoA by CPT2 and then enters β-oxidation followed by the TCA cycle to produce energy. There is evidence that hepatic CPT1 activity is decreased in rats with hypothyroidism and is also a direct target of TH-TR [42,43,44]. Fibroblast growth factor 21 (FGF21), expressed predominantly in the liver, adipose tissue and pancreas, is involved in many metabolic processes, including lipid metabolism. It has been found that transgenic mice overexpressing FGF21 exhibit markedly increased hepatic β-oxidation [45] and reduced hepatic steatosis [46]. Notably, FGF21 is a demonstrated T3-target gene and its regulation is PPARα dependent [46]. 

#### 2.2.4. Cholesterol Homeostasis

Chylomicron remnants (CRs) and lipoproteins (LDL and HDL), another source of liver lipids, are also critical for the maintenance of cholesterol homeostasis by the liver. CRs and lipoproteins enter the liver via LDL receptor (LDLR)- and LDLR-related protein (LRP)-mediated endocytosis [47]. It has long been known that there is a negative correlation between TH and cholesterol in plasma [15], an observation that led to the discovery that levels of LDLRs and LRPs are regulated by THs [48,49]. In addition to being directly regulated at the transcriptional level by THs through TRβ, LDLRs can be indirectly regulated by THs through SREBP2, a key factor in cholesterol biosynthesis in the liver that is transcriptionally regulated by THs [50]. Proprotein convertase subtilisin/kexin type 9 (PCSK9), a secreted protein mainly expressed in the liver, impairs cholesterol uptake by the liver by inducing LDLR degradation [51]. It has been shown that PCSK9 is significantly increased in patients with subclinical hypothyroidism relative to euthyroid controls [52], and that THs and PCSK9 are negatively correlated in humans [52,53]. Therefore, in addition to direct and indirect transcriptional regulation, preventing degradation of LDLR might also be a mechanism by which THs augment cholesterol uptake by the liver. It has further been proposed that THs may regulate plasma cholesterol in an LDLR-independent manner [54]. In mice lacking LDLRs, T3 and TRβ agonists markedly reduce plasma cholesterol by inducing expression of cholesterol 7-α-hydroxylase (CYP7A1) and subsequent promotion of bile acid synthesis. The conversion of cholesterol to bile acid is the major mechanism for clearance of cholesterol by the liver. In an in vitro model, it was shown that the gene encoding CYP7A1, a key enzyme involved in bile acid synthesis, is a direct target of TRβ and is regulated by THs [55]. In addition to increasing the conversion of cholesterol to bile acid, THs can promote biliary elimination of cholesterol. In hypophysectomized rats, administration of T3 ameliorates hypercholesterolemia in association with increased hepatic secretion of cholesterol and upregulation of ABCG5 (ATP-binding cassette, subfamily G, member 5) and ABCG8 [56]. ABCG5 and ABCG8 form a heterodimeric transporter located at the apical plasma membrane of hepatocytes that is responsible for transport of hepatic cholesterol to bile [57]. Studies performed in ABCG5-knockout mice subsequently demonstrated the importance of ABCG5/ABCG8 in biliary cholesterol secretion [58]. In addition to processing cholesterol coming from outside the liver, cholesterol can be synthesized in the liver. HMG-CoA reductase (HMGR) is the rate-limiting enzyme in cholesterol synthesis in the liver, and its expression has been reported to be induced by SREBP2 [59]. As noted above, the gene encoding SREBP2 is a target of THs; thus, THs also affect cholesterol biosynthesis in the liver.

## 3. Pathological Roles of TH Associated with Lipid Metabolism in the Liver

### 3.1. Lipid Metabolism and Liver Diseases: Non-Alcoholic Fatty Liver Disease (NAFLD)

Dysregulation of physiological lipid metabolism is associated with various diseases, such as obesity, diabetes, cardiovascular diseases, and dyslipidemia, among others, that affect different organs [60,61]. Abnormal lipid metabolism in the liver results in the accumulation of excess fat—the so-called fatty liver syndrome; in particular, non-alcoholic fatty liver disease (NAFLD), which is not caused by excess alcohol consumption or viral infection. NAFLD, which affects approximately a quarter of the world’s population, is the most prevalent chronic liver disease worldwide [62]. The pathogenesis of NAFLD is complicated and not fully elucidated. Lifestyle, dietary habits, genetic and epigenetic factors, and microbiota can all result in metabolic dysfunction and contribute to chronic liver disease. 

Liver biopsy and pathological assessment is currently the gold standard for diagnosis of NAFLD, nevertheless, this examination is invasive. As a prescreen, or to avoid invasive testing, NAFLD is usually diagnosed based on imaging and is defined as the presence of at least 5% hepatic steatosis in the context of little or no alcohol consumption or other chronic liver disease [63]. NAFLD can be classified into two types: non-alcoholic fatty liver (NAFL) and non-alcoholic steatohepatitis (NASH). In most patients, NAFLD persists as NAFL, which is considered non-progressive, but about 20% of NAFLD cases progress to NASH [64]. Among the typical features of NASH observed in histological assessments are steatosis, lobular inflammation, and ballooning (hepatocellular injury) [65]. Paik et. al. indicated that, although viral hepatitis remains the leading cause of liver-related death, deaths due to NAFLD are the fastest increasing among non-viral liver-related causes [66]. During the course of NAFLD, a small proportion of individuals will progress to cirrhosis and even hepatocellular carcinoma (HCC) [64]. Nevertheless, there is currently no approved therapy for NAFLD. The increasing prevalence of NAFLD and absence of effective treatment has resulted in NASH becoming the leading cause of liver transplantation in the United State [67]. Both establishment of a prognostic system for NAFLD for identifying the population at high risk of progressing to NASH and the development of therapeutic drugs for NAFLD are currently urgent needs.

### 3.2. Correlations between THs and NAFLD

NAFLD is bidirectionally associated with metabolic diseases such as type 2 diabetes mellitus, dyslipidemia, and hypertension [68], which are also associated with TH dysfunction. Evidence suggests that imbalanced energy metabolism, especially excess carbohydrates and fat delivery into the liver, is a crucial driver of the development of NAFLD [69]. As noted above, THs play crucial roles in energy and metabolic homeostasis; thus, it is reasonable to speculate that THs also play pathogenic roles during the development of NAFLD. Consistent with this, associations between NAFLD and thyroid diseases have been demonstrated. For example, a cross-sectional study of 4648 subjects categorized as controls or patients with subclinical or overt hypothyroidism demonstrated a significant association of NAFLD with hypothyroidism [70]. A higher prevalence of hypothyroidism in patients with NAFLD compared with the control group has also been demonstrated, and patients with hypothyroidism were shown to be more susceptible to NASH [71]. In addition, higher free T4 levels in plasma are associated with a decreased risk of NAFLD [72]. Moreover, in patients diagnosed with NAFLD, low free T3 is independently associated with a high probability of fibrosis and liver stiffness [73]. Collectively, these observations suggest a close association between thyroid function and the development and progression of NAFLD. Nevertheless, discrepant findings have also emerged [74,75,76], possibly owing to variations in the ethnicity of study populations and criteria used for diagnosing hypothyroidism and NAFLD. A large-scale meta-analysis that enrolled 44,140 individuals across 12 cross-sectional and three longitudinal studies revealed that variably defined primary hypothyroidism is significantly and independently associated with the presence and severity of NAFLD, although a causal relationship could not be definitely established. The authors suggested that screening for primary hypothyroidism could be beneficial in distinguishing NAFLD patients who are at high risk of progressing [77]. In sum, multiple lines of evidence support pathological roles of THs in the course of NAFLD, although some conflicting findings remain to be reconciled. Notably, hypothyroidism-induced NAFLD (HIN) is considered a distinct disease entity [78]. Additional large, well-designed studies are warranted to elucidate the relationship between THs and NAFLD and the underlying mechanism linking them.

## 4. Drugs Targeting THs and Their Clinical Applications in NAFLD

### 4.1. Management of NAFLD

In the absence of approved NAFLD-specific treatments, lifestyle modifications with the goal of weight loss, such as improved diet quality and increased physical activity, are the mainstays of treatment for NAFLD [79]. Because visceral obesity promotes insulin resistance and proinflammatory signaling, which are important risk factors for NAFLD, weight loss is directly correlated with substantial improvement in histological outcomes, even fibrosis. For the severely obese population, bariatric surgery may be a therapeutic option [80]. Several drugs developed for other indications have also been tested for potential clinical effects on NAFLD. In one example, pioglitazone, approved for the treatment of type 2 diabetes, combined with vitamin E demonstrated some benefit in randomized trials [81]. However, potent and safe therapeutic strategies specific to NAFLD, the focus of numerous ongoing studies, await discovery.

### 4.2. Therapeutic Potential of THs, TH Mimetics, and TH Metabolites in NAFLD

Given the functions of THs in weight loss and metabolic and energy homeostasis, it is reasonable to anticipate that THs might have potential for the control and treatment of NAFLD. As expected, exogenously administered T3 has been demonstrated to lower hepatic fat content in various experimental animal models of NAFLD [26,82,83]. However, the physiological actions of THs affect almost every organ; thus, administration of THs is accompanied by adverse extrahepatic effects (especially in the heart, muscle, and bone), hampering clinical applications of THs in NAFLD [84]. Expression of TR isoforms is tissue dependent, with TRα being the predominant form expressed in bone and heart and TRβ predominating in the liver [85]. Therefore, compounds that specifically target TRβ were thought to be a solution to the problem of undesired adverse effects. In recent decades, several TRβ-selective agonists have been developed and investigated, including GC-1 and its derivatives, KB2115, MB07811, and MGl-3196 (Table 1), and the potential of several TH metabolites in the treatment of NAFLD have been demonstrated, as detailed below.

#### 4.2.1. GC-1

GC-1, (3,5-dimethyl 1-4(4′-hydroxy-3′-isopropylbenzul- phenoxy) acetic acid), also known as sobetirome, was the first synthetic TR-selective TH analog developed [86]. GC-1 is a shape-mimic of T3 with several structural modifications that weaken the affinity of GC-1 for TRα by ~10-fold but without compromising its affinity for TRβ. In a mouse model of hypothyroidism, GC-1 showed better TG-lowering and similar cholesterol-lowering effects compared with T3 [87]; it also exhibited enhanced hepatic targeting. Both TRβ- and liver-selectivity contribute to the ability of GC-1 to affect lipid metabolism without altering heart rate or weight [87]. The potential of GC-1 to ameliorate NAFLD was reported in several experimental rodent models [26,88]. In a high-fat-diet (HFD) rat model, GC-1 treatment was shown to reduce hepatic TGs by ~75%, but it also caused fasting hyperglycemia and hyperinsulinemia, reflecting a decrease in insulin sensitivity [89]. Similar results were also observed in a study using an ob/ob mice model [88]. These results suggest that, although GC-1 may have potential to treat NAFLD, the effects of GC-1 on insulin sensitivity must be considered.

#### 4.2.2. GC-24

The GC-24, a derivative of GC-1, obtained by modifying the structure of GC-1 exhibits ~40-fold higher binding affinity for TRβ than TRα [90]. In a HFD rat model, administration of GC-24 eliminated the HFD-induced increase in adiposity, increased insulin sensitivity, reduced plasma TG levels, and partially reduced plasma cholesterol levels without causing cardiac hypertrophy. However, hepatic cholesterol content remained high in animals treated with GC-24 [91], which was, subsequently, reported to be less potent against liver targets than GC-1 [92].

#### 4.2.3. KB141

KB141 (3,5-dichloro-4-(4-isopropy acetic acid)), another derivative of GC-1, achieved a ~14-fold higher affinity for TRβ than TRα by virtue of structural modifications [93]. In preclinical animal models, KB141 was shown to increase metabolic rate in mice and rats, and significantly reduce cholesterol, lipoprotein, and body weight in primates [93]. It also has only minimal effects on the heart, but, unlike GC-1, does not preferentially accumulate in the liver [93,94].

#### 4.2.4. KB2115

KB2115 (3-(3,5-dibromo-4-(4-hydroxy-3-(1-methylethyl)-phenoxy)-phenyl)-amino-3-oxopropanoic acid), also known as eprotirome, is a brominated TRβ-selective TH replacement drug that is more liver-specific than GC-1 and has no deleterious effects on the heart [95,97]. Similar to GC-1, administration of KB2115 effectively prevented hepatic steatosis in rodents [88,89]. However, unlike the case for GC-1, administration of KB2115 did not lead to fasting hyperglycemia, although fasting hyperinsulinemia was still noted. A randomized phase 3 study showed that KB2115 lowered cholesterol levels in patients with familial hypercholesterolemia, but also reported evidence of liver injury and cartilage damage [96].

#### 4.2.5. MB07811

MB07811, ((2R,4S)-4-(3-chlorophenyl)-2-((3,5-dimethyl-4-(4′-hydroxy-3′-isopropylbenzyl)phenoxy)methyl)-2-oxido-(1,3,2)-dioxaphosphonane), also known as VK2809, is a prodrug can be processed by hepatic cytochrome P450 enzymes to the active TR agonist, MB07344 ((3,5-dimethyl-4-(4′-hydroxy-3′-isopropylbenzyl)phenoxy) methylphosphonic acid). Ameliorated liver targeting without unfavorable effects on cardiac of MB07811 was demonstrated. Also, administration of MB07811 can reduce hepatic TG levels and also plasma cholesterol and TG [98]. In several experimental models of NAFLD, MB07811 was demonstrated to significantly reduce hepatic steatosis, plasma fatty acids, and TGs without causing liver fibrosis or other liver damage [82]. These experimental findings support the potential of MB0711 for use in treating NAFLD. Two phase 2 clinical trials—NCT02927184 and NCT04173065—have been registered and their results are eagerly awaited.

#### 4.2.6. MGL-3196

MGL-3196 (2-(3,5-dichloro-4-(5-isopropyl-6-oxo-1,6-dihydropyridazin-3-yloxy) phenyl)-3,5-dioxo-2,3,4,5-tetrahydro (1,2,4) triazine-6-carbonitrile), also known as resmetirom, shows a ~28-fold selectivity for TRβ relative to TRα. MGL-3196 lacks adverse cardiac effect and is safe for the central thyroid axis. A study in 53 healthy volunteers showed that MGL-3196 was well-tolerated and decreased LDL cholesterol and TGs without causing adverse reactions [99]. In phase II clinical trials, MGL-3196 reduced LDL cholesterol, TG, and lipoprotein, and, most adverse events noted, including a higher incidence of transient diarrhea and nausea, were mild [100]. Another recent phase II clinical trials also supports the safety and efficacy of MGL-3196 [101], revealing a reduction in LDL cholesterol, apolipoprotein B, TGs, and markers of fibrosis. As of April 2022, there are four ongoing phase 3 trials (NCT03900429, NCT04643795, NCT04951219, and NCT04197479) of MGL-3196 that should provide important data.

#### 4.2.7. 3,5,-L-diiodothyronine (T2)

T2, produced by deiodination of T3 by DIO2, shows ~50–100 times lower affinity for the THR than T3 [102]. T2 was previously considered a mere catabolite of T3, but it has since been demonstrated that T2 can mimic some T3 functions on lipid metabolism. In a HFD rat model, T2 was shown to reduce hepatic fat accumulation, increase the rate of β-oxidation, improve the oxidation-stress status of mitochondria, and reverse liver steatosis without causing notable adverse effects on the heart [103,104]. Subsequent studies revealed that T2 administration prevented HFD-induced insulin resistance [105]. In the same study, the authors suggested that, rather than acting on THRβ, T2 directly activates hepatic nuclear sirtuin 1 (SIRT1), which was previously shown to be capable of modulating lipid metabolism and enhancing mitochondrial activity [106]. In this context, it has been established that direct interactions of SIRT1 with THRβ modulates the expression of T3-regulated hepatic genes [107,108]. T2 acts to regulate lipid metabolism in a different manner compared with T3. Specifically, T2 decreases lipogenesis and increases β-oxidation, whereas the actions of T3 primarily depend on the latter [109]. Although high doses of T2 were found to be associated with serious adverse heart effects [110], overall, most studies have shown beneficial effects of T2 on lipid metabolism, suggesting that T2 is worthy of further study for the prevention and treatment of diseases like obesity and NAFLD.

#### 4.2.8. 3-iodothyronamine (T1AM)

T1AM is another TH metabolite found in blood that is concentrated in some organs, such as the liver, brain, and muscle. Although its biosynthetic pathway is currently unclear, T1AM could theoretically be produced from T4 by enzymatic decarboxylation and deiodination [111]. T1AM mediates its functions through the trace amine-associated receptor (TAAR1) rather than the TR [112]. T1AM was first reported to be an antagonist of T3 [113]; however, recent studies have demonstrated that it induces antilipogenic effects in liver and muscle, and reduces hepatic TG and cholesterol levels in a polycystic ovary rat model [114]. In a spontaneously overweight mouse model, administration of T1AM was shown to increase lipolysis in association with significant weight loss [115]. Additional studies are needed to resolve controversies surrounding the functions of T1AM.

### 4.3. Clinical Application for Thyroid Hormones Status and NAFLD

Thyroid dysfunctions strongly associated with NAFLD and a high prevalence of NAFLD in hypothyroidism patients compared to euthyroid individuals were demonstrated [70,71,72,73,77]. Similarly, the thyroid functions (usually assessed by detecting the levels of serum T3, T4, or TSH) were evaluated in NAFLD patients. However, the results were conflicting. Some studies are showing that circulating T3 or T4 was significantly decreased in NAFLD than that in non-NAFLD [116,117]. The prevalence of hypothyroidism was higher in patients with NAFLD compared to controls [71,118]. In contrast, some studies show inconsistent results [74,119]. Regardless of levels of THs, studies suggest NAFLD patients may acquire resistance to THs [120,121]. All this evidence suggest regulations and actions of THs are extremely complicated and it may be too hasty to conclude using TH replacements or mimetic as an intervention in NAFLD even though close associations between thyroid derangement and NAFLD were established. Fortunately, there are two trials (NCT01848171, and NCT03281083) that started to evaluate the benefits of levothyroxine, the standard TH replacement, on NAFLD in hypothyroidism patients and acquired encouraging results. In phase 4 randomized control trial (NCT01848171) for subclinical hypothyroidism patients revealed the prevalence of NAFLD was significantly reduced after treatment with levothyroxine [122]. Another phase 2b single-arm study (NCT03281083) for euthyroid patients with type 2 diabetes and steatosis revealed that administration of levothyroxine makes a decrease in 12% of baseline intrahepatic lipid content and a small decrease in body mass index, and visceral and subcutaneous adiposity [123]. These results suggest thyroid function should be considered when engaged with NAFLD patients, which may help to find the subpopulations suitable for adjuvant THs analogs therapy to effectively control or even reverse the progression of NAFLD.

Collectively, TH replacement, metabolites, and mimetics show promise for the treatment of NAFLD, however, the safely and efficacy of these compounds need further evaluation in well-designed studies.

## 5. Conclusions and Perspectives

THs are among the most important hormones that regulate multiple physiological activities, including growth, development, and metabolism, throughout life. They remain the focus of research efforts, with numerous studies continuously attempting to more thoroughly understand their functions. Studies on the role of THs in lipid metabolism have revealed their systemic effects in addition to their involvement in intrahepatic pathways. Fully understanding the underlying mechanisms of THs’ actions is challenging. Fortunately, the “omics” field has reached a more mature stage and has become more accessible, providing a powerful tool for elucidating such complex issues. In previous decades, data from small-scale analyses of genes/proteins or comprehensive omics studies, including metabolome studies of blood biochemistry, have most frequently been used to investigate the effects of interventions. More recently, the microbiota/microbiome, especially the gut microbiota/microbiome, has been an emergent topic, with studies demonstrating that gut microbiota influence almost all organs of the body through bidirectional interactions. The microbiota is so thoroughly integrated into human physiology that it is considered an essential organ in humans [124]. Food intake, lifestyle, the microbiota, and human metabolism are all closely related and cannot be considered separately. Future trends for comprehensive studies of metabolic issues are likely to include using “big data” approaches that integrate information from genomic, transcriptomic, proteomic, and metabolomic studies, deep phenotyping, and investigations of the microbiota/microbiome and lifestyle. Such an approach may help unveil the link between thyroid dysfunction and diseases, such as NAFLD, and help to discover new strategies for the prevention, early diagnosis and treatment of NAFLD, and those that improve the prognosis of NAFLD patients. Although such big data applications are still in their infancy, some NAFLD-related studies using this approach have appeared in recent publications [125,126,127].

In addition to the biomarkers and genetic markers, medical imaging technologies have also made great strides in the diagnosis or monitoring of NAFLD. For example, magnetic resonance imaging is considered the gold standard for hepatic fat quantification and shows prominent accuracy to discriminate between different grades of fatty liver [128]. However, innate limitations of medical imaging should be considered when applied to the diagnosis or monitoring of NAFLD. The advantages and limitations of different medical imaging technologies have been summarized elsewhere [129,130]. In sum, the achievements of medical imaging technologies and molecular biologic research could complement each other and help to obtain the best benefit in basic research or clinical application for fatty liver diseases.

The development of thyromimetics and exploration of their potential in the prevention or treatment of NAFLD have continued for decades. Despite these efforts, compounds that combine efficacy and safety have not yet been found. Currently, the main strategy for improving efficacy and reducing adverse effects is to introduce modifications of the base in the backbone of endogenous T3 that increase TRβ- and liver-selectivity. However, the main limit to obtaining a TRβ-specific modification based on the native structure of T3 is the similarity of the ligand-binding domains of TRα and TRβ, which share ~82% homology [86]. In addition, as noted above, metabolism is a complicated systematic issue rather than an issue that involves a single organ. Even if a liver- and TRβ-specific compound is found, extrahepatic factors might interfere with its functions in complicated and unforeseen ways, further increasing the difficulty of research design and limiting the validity and credibility of findings obtained using the current research model. Although many difficulties remain to be overcome, THs clearly have potential in the management of NAFLD and other metabolism-related diseases. Incorporating new research strategies, such as big data, into ongoing and future studies is expected to provide greater insight into the causative relationship between TH function and NAFLD and other related diseases.

## Figures and Tables

**Table 1:** Summary of thyromimetics.

Compound	Type	Favorable Effects	Unfavorable Effects	Clinical Trials
GC-1 (Sobetirome)	TRβ- and liver- selective thyromimetic	10-fold lower TRα affinity [86] ↓ triglyceride [87,88] ↓ cholesterol [87] Prevent hepatic steatosis in rodent model [26,88,89] ↑ β-oxidation [26]	Fasting hyperglycemia and hyper- insulinemia [89]	
GC-24	TRβ-selective thyromimetic	40-fold higher TRβ affinity than TRα [90] ↑ energy expenditure [91] Eliminate the increase in adiposity [91] ↑ insulin sensitivity [91] ↓ triglyceride [91]	No improve in hepatic cholesterol [91] Less potent on liver target [92]	
KB141	TRβ-selective thyromimetics	14-fold higher affinity to TRβ than TRα [93] ↑ metabolic rate [93] ↓ cholesterol [93] ↓ lipoprotein [93] ↓ body weight [93]	↑ heart rate [93] No liver- selective [93,94]	
KB-2115 (Eprotirome)	TRβ- and liver- selective thyromimetic	↓ triglyceride [88] Ameliorate hepatic steatosis [88,89] ↓ LDL cholesterol [95] ↑ bile acid synthesis [95]	Fasting hyperinsulinemia [89] Liver injury and cartilage damage [96]	NCT00593047 (phase 2) [97] NCT00776321 (phase 2) NCT00677248 (phase 2) NCT01410383 (phase 3) [96]
MB07811 (VK2809)	TRβ- and liver-selective prodrug	↓ cholesterol [98] ↓ triglyceride [82,98] ↓ hepatic steatosis [82] ↓ free fatty acid [82] ↑ β-oxidation [82]		NCT02927184 (phase 2) NCT04173065 (phase 2)
MGL-3196 (Resmetirom)	TRβ- and liver- selective thyromimetic	28-fold higher affinity to TRβ than TRα [99] ↓ cholesterol [99,100] ↓ triglyceride [99,100] ↓ lipoprotein [100] ↓ fibrosis [100]	Mild diarrhea and nausea [100]	NCT03900429 (phase 3) [101] NCT04643795 (phase 1) NCT04951219 (phase 3) NCT04197479 (phase 3)

## Data Availability

Not applicable.
